# Deep Vein Thrombosis in a 15-Year-Old Previously Restrained Psychiatric Patient: A Case Report

**DOI:** 10.7759/cureus.62261

**Published:** 2024-06-12

**Authors:** Marcin Mikusek-Pham Van

**Affiliations:** 1 Department of Child Psychiatry, University Clinical Center, Warsaw, POL

**Keywords:** complication, direct coercion, restraint, deep vein thrombosis, child and adolescent psychiatry

## Abstract

Deep vein thrombosis is a condition in which a thrombus forms in one of the deep veins of the body, most often in the legs. It may manifest with pain, swelling, redness, or increased temperature of the limb, potentially leading to fatal complications such as pulmonary embolism. This is a case of a 15-year-old psychiatric patient diagnosed with deep vein thrombosis of the left lower limb of uncertain etiology. The patient presented few risk factors associated with venous thromboembolism disorder such as immobilization, antipsychotic treatment, and obesity. Even though psychiatry deals primarily with the mind of a patient, somatic complications occur very often and should not be underestimated. One of those complications is deep vein thrombosis, which is worth remembering, especially when applying procedures during which patients are immobilized for a long period.

## Introduction

Deep vein thrombosis (DVT) is a medical condition characterized by the formation of blood clots in the deep veins, predominantly affecting legs (calf, femoral, or popliteal veins); however, it can also occur in the deep veins of the pelvis or arms [1]. Thrombi can lead to serious complications, including pulmonary embolism (PE) and post-thrombotic syndrome, characterized by chronic pain, swelling, skin induration, and venous ectasia [2]. DVT combined with PE creates venous thromboembolism (VTE) disorder, which occurs in around 1.6 per 1,000 persons yearly [3]. Approximately two-thirds of patients with VTE present with DVT, while one-third present with PE [4]. Around 6% of DVT and 12% of PE patients die within one month of diagnosis [3]. Interestingly, DVT incidence rises significantly with age, affecting mostly people over the age of 40 [5,6]. However, two peaks of DVT in children have been identified: the neonatal period and adolescence [7]. The risk of DVT can be heightened by several factors such as prolonged immobilization [8], increased venous pressure [3], high blood viscosity [9], and genetic deficiencies of human anticoagulants [10].

Signs and symptoms of DVT involve, among others, pain, edema, fever, tenderness, and erythema. Unfortunately, around 50% of patients with acute DVT can be asymptomatic, highlighting the need for more objective diagnostic techniques [11]. Currently, the diagnosis of DVT involves the well-studied Wells score [12], D-dimer levels, and Doppler ultrasonography [3]. Prevention and treatment typically consist of anticoagulants such as heparin and warfarin, although newer direct oral anticoagulants, such as rivaroxaban and apixaban, are also used [13]. Here, I present the case of a psychiatric patient diagnosed with DVT with few predisposing risk factors.

## Case presentation

A 15-year-old girl was admitted to a child psychiatry ward on October 11, 2021, previously diagnosed with spastic diplegic cerebral palsy (characterized by increased tendon reflex activity and hypertonia), mild intellectual disability, and obesity. Prior to the hospitalization, the patient did not require any mobility aid. She was diagnosed with thrombophlebitis of the lower extremities on September 20, 2021. At the time of admission, she weighed 75.5 kg (98th percentile), was 159.5 cm tall (27th percentile), and had a BMI of 29.7 kg/m^2^ (99th percentile). General laboratory tests performed at the time of admission presented no significant abnormalities (Table 1).

**Table 1:** Laboratory tests on admission ALT, alanine aminotransferase; AST, aspartate aminotransferase; CRP, C-reactive protein; HCT, hematocrit; HGB, hemoglobin; MCH, mean corpuscular hemoglobin; MCHC, mean corpuscular hemoglobin concentration; MCV, mean corpuscular volume; PLT, platelet; RBC, red blood cell; WBC, white blood cell

Laboratory parameters	Result	Reference range
WBC (10^3^/μL)	4.76	4.0-10.0
RBC (10^6^/μL)	4.31	4.2-5.4
PLT (10^3^/μL)	276	150-400
HCT (%)	36.6	37.0-47.0
HGB (g/dL)	12.4	12.0-16.0
Lymphocytes (10^3^/μL)	1.99	1.5-3.9
Neutrophils (10^3^/μL)	2.06	2.5-6
Basophils (10^3^/μL)	0.03	<0.1
Monocytes (10^3^/μL)	0.48	0.18-0.78
Eosinophils (10^3^/μL)	0.19	<0.44
MCH (pg)	28.8	26.0-32.0
MCHC (%)	33.9	31.0-35.0
MCV (fL)	84.9	78.0-95.0
ALT (U/I)	27	<47
AST (U/I)	23	<46
Creatinine (mg/dL)	0.67	0.5-0.9
CRP (mg/dL)	0.57	<0.5

About 1.5 months before the admission to the department, the patient presented impaired consciousness, slowness of speech, psychomotor agitation, and non-bizarre delusions (assessed by a pediatric neurologist). Before the onset of symptoms, the girl experienced periods of insomnia lasting up to 30-40 hours. As a result, she was referred to the Neuropsychiatric Center on August 31, 2021. During the hospitalization, she received antipsychotic drugs (risperidone, levomepromazine, haloperidol) and benzodiazepines (lorazepam, clorazepate). Ultimately, the patient was treated with risperidone (2 mg/d) and lorazepam (4 mg/d). The remaining medications were administered as needed. She was subjected to direct coercion several times due to her life-threatening behavior. The patient also suffered from pneumonia and spent most of her time in the hospital bed. She was treated with amoxicillin/clavulanic acid for 10 days, and it had a good effect. In the third week, the patient reported pain in the left lower limb, which was the only symptom she reported. Doppler ultrasound was performed; the patient was diagnosed with DVT and referred to our hospital for comprehensive care. Initially, the patient stayed at the surgical and later at the neurological ward in order to stabilize her somatic state (from September 20 to October 11, 2021). Initial D-dimer and fibrinogen levels were 60,606 ng/mL and 4.76 g/L, respectively (Table 2). Doppler ultrasonography of the left lower limb revealed that the common iliac vein, external iliac vein, femoral vein, and deep femoral vein were non-compressible and filled with thrombi. The patient underwent chest computed tomography (CT), and PE was ruled out. Because of her deteriorating mental state and disturbances of consciousness, a CT of the brain was performed, which did not reveal any clinically significant changes. The patient received 180 mg/d of enoxaparin for three weeks (since September 21, 2021), then rivaroxaban 30 mg/d for three weeks, and later 20 mg/d for three months.

**Table 2:** Evolution of D-dimer and fibrinogen levels throughout the hospitalization

Hospitalization day	Day 2	Day 3	Day 10	Day 16	Day 26	Day 33	Day 39	Day 73	Day 122	Reference value
D-dimer (ng/mL)	60,606	10,830	10,028	4,869	1,892	1,306	1,117	994	352	<550
Fibrinogen (g/L)	4.76	5.60	4.71	3.52	3.68	3.64	3.53	3.55	3.77	1.5-3.5

After the patient was stabilized, she was transferred to the child psychiatry ward for further treatment and in-depth psychiatric diagnosis. Previously used psychiatric medications (risperidone and lorazepam) were discontinued. The psychiatric diagnosis of bipolar affective disorder (currently mixed episodes) was made. Carbamazepine (400 mg in the morning, 300 mg in the evening) and chlorpromazine (25.8 mg in the morning, 51.6 mg in the evening) were introduced, resulting in a reduction of aggressive behavior, disappearance of psychotic symptoms, and mood stabilization. Moreover, other symptoms, such as slowness of speech, impaired consciousness, and insomnia, also disappeared. Anticoagulant treatment was continued in the department and had a good effect. The last known D-dimer and fibrinogen levels were 352 ng/mL and 3.77 g/L, respectively. She was discharged after almost four months of stay (on February 8, 2022) in good general condition with a recommendation for further outpatient care. During the follow-up, the patient continued the treatment introduced in the child psychiatry department and did not suffer from any additional thromboembolic complications.

## Discussion

DVT represents a serious complication in psychiatric settings, particularly concerning patients subjected to direct coercive measures. In the case of our patient, there were several predisposing factors. First of all, the patient was subjected to physical restraint. A retrospective analysis of 1,308 psychiatric inpatients has shown that patients who are physically restrained are at a higher risk for developing DVT (OR = 6.0, p < 0.01). This is attributed to immobilization, which impedes venous blood flow, possibly aggravating the clotting cascade [14]. Moreover, our patient received antipsychotics. A population-based control study found that individuals who received antipsychotic drugs in the previous 24 months had a 32% greater risk of VTE in comparison to non-users (OR = 1.32, 95% confidence interval). The risk was particularly high in new users and those receiving atypical antipsychotics [15]. Certain drugs, especially second-generation antipsychotics, are strongly associated with VTE. The highest number of VTE cases were reported in clozapine patients, followed by risperidone and olanzapine [16]. Interestingly, Ishida et al. pointed out several risk factors associated with a higher risk of DVT in restrained psychiatric patients: longer duration of restraint (>24 hours), excessive sedation, and, most importantly, lower antipsychotic dosage: <600 mg/d of chlorpromazine equivalents (OR = 0.05, p = 0.016) [17]. This leads to a paradox in which antipsychotics’ risk of DVT generally increases with dose [18]; however, this relationship does not apply to patients subjected to restraint, posing the need for further research. Another factor worth paying attention to is obesity (the patient was in the 99th percentile in terms of body weight). The risk of DVT rises with increased BMI; the hazard ratio for moderately obese was 1.8, and for severely obese, 3.4 [19]. Moreover, our patient suffered from a specific form of cerebral palsy, spastic diplegia, which often results in limited mobility and prolonged bedrest, leading to venous stasis, a major risk factor for DVT [3].

The diagnosis of DVT in psychiatric patients can be challenging. Because of their psychiatric issues and the sedative effect of received medication, they might be unaware of somatic symptoms [20]. Prophylactic interventions, such as the use of anticoagulants and graduated compression stockings, are essential yet require careful application to balance the benefits against potential risks like bleeding. Effective prophylaxis includes regular screening and quick mobilization if possible [17].

## Conclusions

Physical restraint is considered a last resort method to control an aggressive patient. However, like any medical procedure, it carries the risk of complications. One of such complications is DVT, which should be especially remembered in patients with risk factors such as obesity, antipsychotic drug treatment, or in patients immobilized for a long period due to direct coercion or an underlying condition like spastic diplegia cerebral palsy. Therefore, if a patient reports pain, swelling, or redness of a limb, thorough diagnostics should be performed to exclude this potentially life-threatening condition. Given the dangerous complications associated with direct coercion, medical departments are encouraged to revise their protocols. Avoiding the use of physical restraint, seeking safer alternatives, and administering routine thromboprophylaxis for high-risk patients are recommended strategies to mitigate the incidence of DVT.

## References

[1] Kesieme E, Kesieme C, Jebbin N, Irekpita E, Dongo A (2011). Deep vein thrombosis: a clinical review. J Blood Med.

[2] Kahn SR, Solymoss S, Lamping DL, Abenhaim L (2000). Long-term outcomes after deep vein thrombosis: postphlebitic syndrome and quality of life. J Gen Intern Med.

[3] Waheed SM, Kudaravalli P, Hotwagner DT (2024). Deep Vein Thrombosis. StatPearls. Treasure Island.

[4] Nielsen JD (2013). The incidence of pulmonary embolism during deep vein thrombosis. Phlebology.

[5] Huang Y, Ge H, Wang X, Zhang X (2022). Association between blood lipid levels and lower extremity deep venous thrombosis: a population-based cohort study. Clin Appl Thromb Hemost.

[6] Silverstein MD, Heit JA, Mohr DN, Petterson TM, O'Fallon WM, Melton LJ 3rd (1998). Trends in the incidence of deep vein thrombosis and pulmonary embolism: a 25-year population-based study. Arch Intern Med.

[7] Parasuraman S, Goldhaber SZ (2006). Venous thromboembolism in children. Circulation.

[8] Kearon C (2003). Natural history of venous thromboembolism. Circulation.

[9] Vayá A, Suescun M (2013). Hemorheological parameters as independent predictors of venous thromboembolism. Clin Hemorheol Microcirc.

[10] Senst B, Tadi P, Basit H, Jan A (2024). Hypercoagulability. StatPearls. Treasure Island.

[11] Min SK, Kim YH, Joh JH (2016). Diagnosis and treatment of lower extremity deep vein thrombosis: Korean practice guidelines. Vasc Specialist Int.

[12] Wells PS, Anderson DR, Bormanis J (1997). Value of assessment of pretest probability of deep-vein thrombosis in clinical management. Lancet.

[13] Hirsh J, Hoak J (1996). Management of deep vein thrombosis and pulmonary embolism. A statement for healthcare professionals. Council on Thrombosis (in consultation with the Council on Cardiovascular Radiology), American Heart Association. Circulation.

[14] Funayama M, Takata T (2020). Psychiatric inpatients subjected to physical restraint have a higher risk of deep vein thrombosis and aspiration pneumonia. Gen Hosp Psychiatry.

[15] Parker C, Coupland C, Hippisley-Cox J (2010). Antipsychotic drugs and risk of venous thromboembolism: nested case-control study. BMJ.

[16] Shulman M, Jennifer Njoku I, Manu P (2013). Thrombotic complications of treatment with antipsychotic drugs. Minerva Med.

[17] Ishida T, Katagiri T, Uchida H, Takeuchi H, Sakurai H, Watanabe K, Mimura M (2014). Incidence of deep vein thrombosis in restrained psychiatric patients. Psychosomatics.

[18] Masopust J, Malý R, Vališ M (2012). Risk of venous thromboembolism during treatment with antipsychotic agents. Psychiatry Clin Neurosci.

[19] Klovaite J, Benn M, Nordestgaard BG (2015). Obesity as a causal risk factor for deep venous thrombosis: a Mendelian randomization study. J Intern Med.

[20] Dickson BC, Pollanen MS (2009). Fatal thromboembolic disease: a risk in physically restrained psychiatric patients. J Forensic Leg Med.

